# Leptomeningeal carcinomatosis associated with papillary renal cell carcinoma

**DOI:** 10.3332/ecancer.2014.468

**Published:** 2014-10-02

**Authors:** Abhishek Chilkulwar, Ramya Pottimutyapu, Fawng Wu, Keerthi R Padooru, Sai Ravi Pingali, Mohammed Kassem

**Affiliations:** 1 Department of Haematology and Oncology, Allegheny General Hospital, Pittsburgh, PA 15212, USA; 2 Department of Internal Medicine, Mount Sinai Hospital Medical Centre, Chicago, Illinois 60608, USA; 3 Department of Medicine, S.V.S Medical College, Mahbub Nagar, Telangana 509001, India; 4 Houston Methodist Cancer Centre, 6445 Main St, Houston, TX 77030, USA

**Keywords:** papillary renal cell carcinoma, leptomeningeal metastasis, neoplastic meningitis, leptomeningeal carcinomatosis

## Abstract

Leptomeningeal carcinomatosis (LMC), or neoplastic meningitis, occurs in about 5–20% of patients with metastatic cancer, depending on the type of the primary malignancy and kind of treatment received. The association of LMC with renal cell carcinoma (RCC) is a rare entity, and only two cases of papillary renal cell cancer with leptomeningeal metastasis have been reported. Leptomeningeal spread usually confers a poor prognosis despite the use of modern treatment strategies as compared to patients with extracranial metastases. We report a case of papillary RCC, a less common type of RCC presenting with LMC.

## Introduction

Cancers of the kidney and renal pelvis account for approximately 3% of all cancers in the United States [1]. Renal cell carcinoma (RCC) accounts for about 7% of all cancers in men. There are several subtypes of RCC, of which clear cell carcinoma accounts for about 70–80% of the cases, followed by papillary carcinoma/chromophil (10–15%) and chromophobe (5%) [2].

Haematuria and abdominal pain are the most common presenting symptoms of RCC. It can also present as a flank mass, scrotal varicocele, or metastatic disease with involvement of the lymph nodes, lungs, liver, bones, and brain. Nearly 60% of RCCs are incidentally detected in persons with no genitourinary symptoms. Unfortunately, most RCCs remain clinically dormant for a long time until they are locally advanced, metastasised, or unresectable. The prognosis in such advanced cases is generally poor [2].

Leptomeningeal carcinomatosis (LMC), also known as neoplastic meningitis (NM), denotes the spread of the tumour to the meninges with or without parenchymal involvement. LMC is common with solid tumours, such as those of the lung and breast, and also with haematological malignancies like acute lymphoid leukaemia and lymphomas. While metastasis of RCC to the brain parenchyma is common, very few cases of leptomeningeal involvement have been described in the literature. The significance of this rare presentation lies in the fact that it usually indicates poor prognosis and is challenging to treat. Here, we present a rare case of papillary RCC with LMC.

## Case report

**Figure 1.**
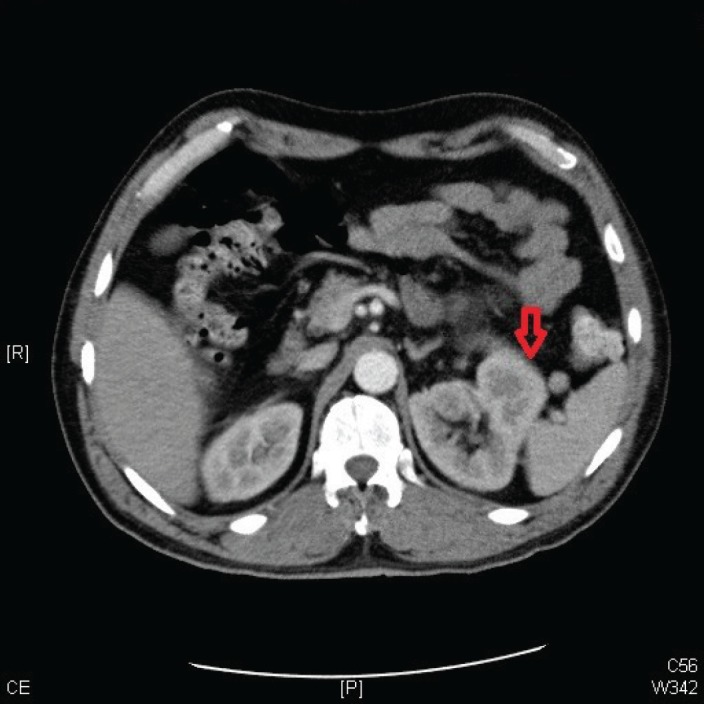
A CT scan of the abdomen and pelvis showing a left renal mass 13 cm in diameter with features suggestive of primary RCC.

A 57-year-old man presented to his primary care physician’s office with haematuria of one-month duration. He also reported a 10–15 lb weight loss over the preceding 4–6 weeks. A review of symptoms was positive for anorexia and lower-back pain for the preceding two months. Urinalysis confirmed the presence of haematuria without evidence of any infectious process. A computed tomography (CT) scan of the abdomen and pelvis revealed a left renal mass (measuring 13 cm in diameter) with imaging characteristics suggestive of primary RCC (Figure 1). The workup for metastatic diseases with magnetic resonance imaging (MRI) of the brain, CT of the chest, and a bone scan was negative. The patient was evaluated by urology and was scheduled for nephrectomy but had to return to the hospital a week sooner than scheduled because of symptoms of right-flank pain, worsening back pain, and progressive weakness of both legs. The lower-extremity weakness started a week prior to presentation and progressively got worse, resulting in the inability to walk. The weakness was associated with urinary incontinence. The rest of the review of symptoms was negative.

The past medical history was significant for hypertension. He denied any significant family history. His personal history was significant for a 15 to 20 pack per year history of smoking.

On physical examination, the patient had tenderness in the lower back and over the right costovertebral angle. Motor power was 5/5 in both upper extremities, 3/5 in the left lower extremity, and 2/5 in the right lower extremity, in both the proximal and distal muscle groups. The gait could not be tested because of the patient’s inability to stand up. There was decreased anal sphincter tone, and sensation was impaired in the perineal region. There was no evidence of meningimus, and the cranial nerve examination was normal.

**Figure 2.**
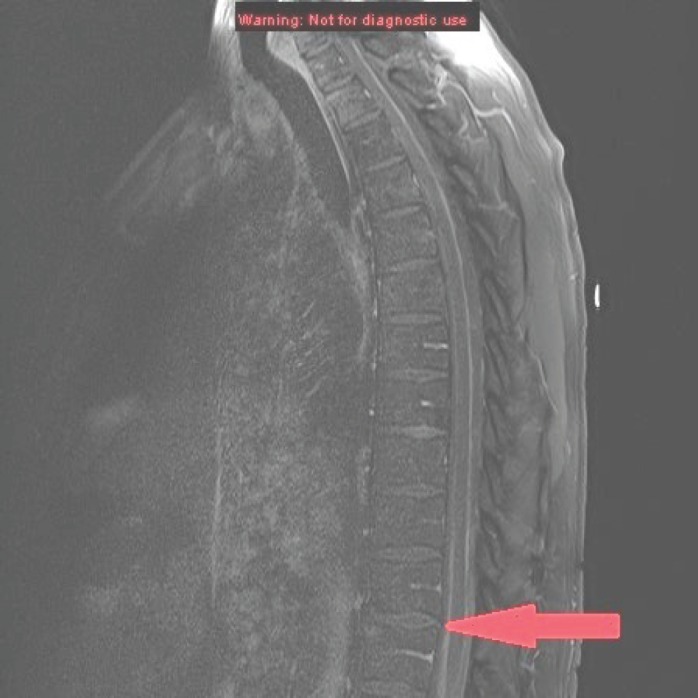
MRI of the spine sagittal section showing anterior and posterior parallel thick lines of avid enhancement corresponding to the leptomeninges, which is highly abnormal and indicates leptomeningeal carcinomatosis.

**Figure 3.**
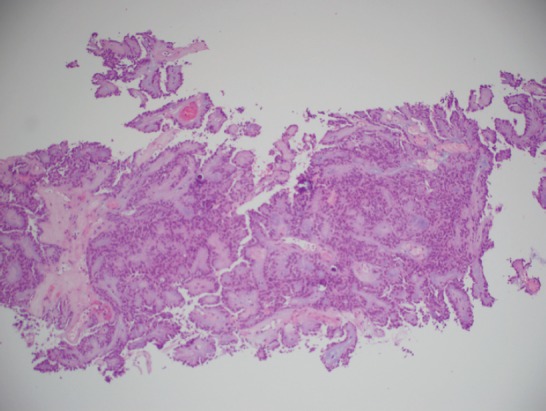
A histopathological exam confirming papillary RCC.

**Figure 4.**
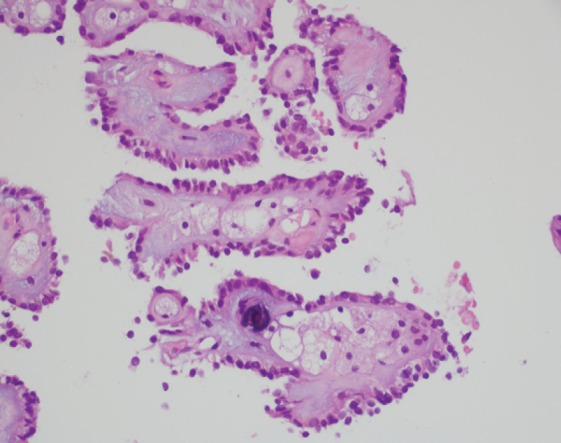
A histopathological exam confirming papillary RCC.

MRI of the spine demonstrated nodular enhancement of the spinal cord with diffuse leptomeningeal involvement along with osseous metastasis (Figure 2). MRI of the brain was done and showed a mildly increased signal within the sulci of the posterior fossa, raising suspicion for leptomeningeal metastasis. A CT-guided biopsy of the nodular lesion at L3–L4, along with a spinal tap, was performed. While the biopsy of the nodular lesion was inconclusive, the cerebrospinal fluid (CSF) cytology was positive for numerous atypical cells consistent with metastatic carcinoma. The patient was treated with radiation therapy to the spinal cord and high-dose steroids. Since the patient was not a surgical candidate because of diffuse leptomeningeal involvement, he underwent further workup with biopsy of the renal mas which showed nonclear cells with immunophenotype staining positive for racemase, ck7, and negative for CD10, consistent with papillary sub-type of RCC (Figures 3 and 4).

The patient received a total of 30 Gy of craniospinal irradiation along with steroids with only a mild improvement in symptoms. Because of the paucity of data for nonclear cell carcinoma and to an even greater extent for leptomeningeal metastasis, a literature review was carried out, and the patient was started on temsirolimus therapy and discharged home with physical therapy. The patient underwent outpatient treatment with temsirolimus for three cycles. After the third cycle of chemotherapy, the patient returned to the hospital with worsening leg weakness, altered mental status, and declining performance status. He progressed from Eastern Cooperative Oncology Group (ECOG) performance status grade 1 to grade 3. A CT scan of the head showed hydrocephalus, secondary to worsening metastatic lesions, for which he underwent placement of a ventriculoperitoneal shunt. The patient’s hospital course was further complicated by the syndrome of inappropriate antidiuretic hormone secretion (SIADH) and hyponatremia. Given the patient’s clinical deterioration, he was discharged with home hospice care as per the family’s wishes.

## Discussion

LMC or NM occurs in about 5–20% of patients with metastatic cancer. There has been a recent rise in the diagnosis of leptomeningeal metastasis partly because of increased survival and also because of aggressive diagnostic modalities in pursuit of the diagnosis [3–5]. Association of LMC with RCC is a rare entity, with very few cases reported in the literature. The most common solid organ tumour associated with LMC is breast cancer, which accounts for about 30% of cases followed by lung (25%) and melanoma (20%). Haematopoietic cancers like lymphoma (7–15%) and leukaemia (5–15%) also contribute to a significant number of cases of leptomeningeal metastasis. Primary brain tumours contribute to about 1–2% of cases [3–8].

LMC can be caused by haematogenous spread, direct extension from dural or parenchymal metastasis, and/or spread from the venous plexus (from leptomeningeal veins). Haematogenous spread is the most common route. Perineural extension along the epineurium or even along the perineurium of cranial or spinal nerves can occur, particularly from paravertebral metastases. Once the cells reach the leptomeningeal space, there can be diffuse involvement because of the flow of CSF. Increased cell adhesion can lead to the formation of bulky masses. Even in the absence of gross evidence of disease, there can be microscopic evidence of disseminated tumour involvement of the leptomeninges throughout the central nervous system [3–8].

Multifocal involvement is a hallmark of diffuse leptomeningeal disease. Patients may present with many neurologic signs, depending upon the site of leptomeningeal invasion. Signs associated with meningismus and/or increased intracranial pressure are very common, as noted in our patient. Brain parenchymal involvement can be focal, with signs or symptoms mimicking stroke, or diffuse, with mental status changes, generalised seizures, and encephalopathy. Diabetes insipidus or panhypopituitarism, though rare, can occur because of leptomeningeal invasion of the pituitary stalk. Patients can also present with radicular symptoms along with bowel and bladder incontinence resulting from involvement of spinal nerve roots and spinal cord, as seen in our patient [3–7, 9, 10].

A careful history, thorough neurologic examination, imaging studies, and CSF evaluation is imperative for accurate diagnosis. Although the sensitivity of diagnostic techniques has improved over time, the diagnosis of leptomeningeal metastasis remains challenging, and neither CSF cytology nor MRI is adequately sensitive by itself. The first diagnostic test performed in a patient with suspected leptomengial metastasis should be MRI of the brain and full spinal cord. Leptomeningeal enhancement in leptomengial metastasis can be linear but often has irregularity or nodularity associated with it [7]. MRI used by itself has a high rate of false negativity (about 30–60%), but in the presence of classic signs and symptoms, MRI by itself may be adequate enough to make the diagnosis. Lumbar puncture is less sensitive than MRI but is more specific. The sensitivity of lumbar puncture can be increased when repeated multiple times. Sensitivity reaches up to 80% when lumbar puncture is repeated three times. Lumbar puncture is 100% specific, and a positive CSF cytology establishes the diagnosis of LMC. The CSF shows a high-opening pressure with elevated protein and low glucose. MRI and CSF analysis are complementary, and the use of both these increases diagnostic accuracy [3–11]. However, negative MRI and negative CSF analysis does not exclude the diagnosis of leptomeningeal metastasis.

Tumour markers (e.g., CEA, PSA, CA-15-3, CA-125, and MART-1, and MAGE-3 in melanoma) may provide evidence for CSF dissemination of disease, even when serial cytological evaluations are negative. Immunohistochemical staining of cells in the CSF may provide diagnostic information or suggest the primary site in a patient with an unknown primary. CSF flow cytometry or molecular studies may be particularly valuable in diagnosing LM from haematologic malignancies [7, 8].

## Conclusion

As is true with any metastasis, NM or LMC indicates a very poor prognosis and excess tumour burden. The median survival of a patient with untreated leptomeningeal metastasis is around 4–6 weeks, and therapy is mainly aimed at improving the quality of life and neurological deficits and preventing disease progression [3, 6–8]. Its association with RCC is yet to be established, as there are relatively very few data. Needless to say, the rarity of this presentation creates a major medical dilemma with treatment options of chemotherapy versus radiation versus surgery, or a combination of all these modalities. Given the poor outcomes in patients with leptomeningeal metastasis, there is a need for further studies to develop treatment regimens that reduce the disease burden and improve quality of life.

## Sources of support

None.

## Conflicts of interest

The authors have no conflicts of interest to declare.
